# Sleep and respiratory abnormalities in adults with developmental and epileptic encephalopathies using polysomnography and video‐EEG monitoring

**DOI:** 10.1002/epi4.12772

**Published:** 2023-06-12

**Authors:** Shobi Sivathamboo, Kenneth A. Myers, Andreas Pattichis, Elise J. White, Ka Nyuk Ku, Terence J. O'Brien, Piero Perucca, Patrick Kwan

**Affiliations:** ^1^ Department of Neuroscience, Central Clinical School Monash University Melbourne Victoria Australia; ^2^ Department of Medicine (The Royal Melbourne Hospital) The University of Melbourne Parkville Victoria Australia; ^3^ Department of Neurology The Royal Melbourne Hospital Parkville Victoria Australia; ^4^ Department of Neurology Alfred Health Melbourne Victoria Australia; ^5^ Research Institute of the McGill University Health Centre Montreal Quebec Canada; ^6^ Department of Pediatrics, Montreal Children's Hospital McGill University Montreal Quebec Canada; ^7^ Department of Neurology and Neurosurgery, Montreal Children's Hospital McGill University Montreal Quebec Canada; ^8^ Department of Respiratory Medicine Royal Melbourne Hospital Melbourne Victoria Australia; ^9^ Department of Respiratory Medicine Alfred Health Melbourne Victoria Australia; ^10^ Bladin‐Berkovic Comprehensive Epilepsy Program Austin Health Heidelberg Victoria Australia; ^11^ Epilepsy Research Centre, Department of Medicine (Austin Health) The University of Melbourne Heidelberg Victoria Australia

**Keywords:** DEE, epilepsy, sleep‐disordered breathing

## Abstract

This study evaluated sleep and respiratory abnormalities, and their relationship with seizures, in adults with developmental and epileptic encephalopathies (DEEs). We studied consecutive adults with DEEs undergoing inpatient video‐EEG monitoring and concurrent polysomnography between December 2011 and July 2022. Thirteen patients with DEEs were included (median age: 31 years, range: 20–50; 69.2% female): Lennox–Gastaut syndrome (*n* = 6), Lennox–Gastaut syndrome‐like phenotype (*n* = 2), Landau–Kleffner syndrome (*n* = 1), epilepsy with myoclonic‐atonic seizures (*n* = 1), and unclassified DEEs (*n* = 3). Sleep architecture was often fragmented by epileptiform discharges and seizures resulting in arousals (median arousal index: 29.0 per h, range: 5.1–65.3). Moderate‐to‐severe obstructive sleep apnea (OSA) was observed in seven patients (53.8%). Three patients (23.1%) had tonic seizures that frequently occurred with central apnea; one met criteria for mild central sleep apnea. Of the patients with tonic seizures, two had other identifiable seizure manifestations, but in one patient, central apnea was commonly the only discernable seizure manifestation. Polysomnography during video‐EEG is an effective diagnostic tool in detecting sleep and seizure‐related respiratory abnormalities. Clinically significant OSA may increase the risk of comorbid cardiovascular disease and premature mortality. Treatment of epilepsy may improve sleep quality, and conversely, improved sleep, may decrease seizure burden.

## 1 INTRODUCTION

Developmental and epileptic encephalopathies (DEEs) are a group of heterogenous neurodevelopmental disorders, characterized by early onset seizures, abnormal electroencephalographic (EEG) patterns, developmental delay or regression, and impaired behavioral function.^1^ In this group of patients, the underlying epileptiform activity contributes to severe cognitive and behavioral impairments, beyond what would be expected from the underlying pathology alone.^1^ Many DEEs are marked by distinct and frequent epileptiform abnormalities and seizures occurring in sleep, which may affect brain networks that regulate sleep and breathing.^2^, ^3^ The frequency and types of sleep disorders in this population, however, remains poorly understood.

Screening instruments completed by caregivers report a high prevalence of sleep disorders in patients with DEEs.^4^, ^5^ However, these instruments have limited reliability in this population and more objective measurements to assess the relationship between sleep and EEG abnormalities are needed. Existing studies using overnight polysomnography or oximetry in individuals with DEEs have been limited by sample size, and have exclusively studied pediatric patients.^4^, ^6^ These investigations suggest potential alterations to sleep architecture,^6^ and frequent nocturnal oxygen desaturations^4^; however, published data are conflicting.^7^

To the best of our knowledge, there have been limited polysomnography studies of adults with DEEs who may have additional risk factors for sleep disorders. In this study, we examined sleep and EEG abnormalities in adults with DEEs using polysomnography during video‐EEG monitoring.

## 2 MATERIALS AND METHODS

### 2.1 Study participants

This prospective cohort study identified adults (aged ≥18 years) diagnosed with a DEE during admission for inpatient video‐EEG monitoring at The Royal Melbourne and Alfred hospitals, Australia, between December 2011 and July 2022.

Reason for referral primarily included diagnostic evaluation for a potential uncontrolled seizure disorder, or to undergo evaluation for epilepsy surgery or neuromodulation therapy. Patient history, neurological examination, EEG recordings, neuropsychiatric evaluation, neuropsychological assessment, and neuroimaging including MRI, and SPECT or PET as appropriate, were considered in the final diagnosis. All changes to ASM therapy were reviewed at the end of the admission from a multidisciplinary reviewing consisting of at least ≥2 epileptologists and were initiated at discharge. Patients diagnosed with DEE at the end of the admission according to the 2017 International League Against Epilepsy (ILAE) definition were included.^1^ Specific epilepsy syndrome diagnosis was made based on the 2022 ILAE classification.^8^ Detailed methodology has been previously published.^9^

Data were collected regarding demographics and anthropomorphic measurements (height, weight, body mass index, and neck circumference). A detailed medical history including information regarding seizure or event semiology and frequency, age of disease onset, current ASM treatment, other medication use, and cardiopulmonary history was collected at the time of admission from interview with caregivers or review of medical records.

### 2.2 Polysomnography

The overnight polysomnography was routinely conducted on the fourth evening of the video‐EEG monitoring, irrespective of history of sleep disturbance.^9^, ^10^ A minimum of 21‐EEG scalp electrodes were applied in accordance with the 10–20 International System of Electrode Placement. Lead II of a standard 12 lead ECG was used for cardiac monitoring. Respiratory inductance plethysmography thoracic and abdominal bands, pulse oximetry to assess peripheral capillary oxygen saturation levels, nasal pressure, electrooculography, electromyography electrodes to the mentalis and sub‐mentalis, and dual electromyography electrodes to each anterior tibialis were added. All measurements were recorded using Compumedics ProFusion 5 (Compumedics Limited). The polysomnography was set up by a qualified sleep scientist and monitored during the night by trained nursing staff.

All sleep studies were reported by an accredited sleep and neurophysiology scientist (E.W. or K.K.) and board‐certified neurologist and sleep/respiratory physician (A.P.). Sleep staging and respiratory event scoring was in accordance with the latest guidelines.^11^ Apneas were scored when there was a ≥90% reduction in airflow amplitude for ≥10 s. Hypopneas were scored where there was a ≥30% reduction in airflow amplitude for ≥10 s, accompanied by either an arousal or desaturation of ≥3%. Data that were missing, unavailable, or obscured by artifact during the polysomnography were not considered in the final generation of the apnea–hypopnea index (AHI). We defined sleep‐disordered breathing as an apnea–hypopnea index (AHI) ≥5, and moderate‐to‐severe sleep‐disordered breathing as an AHI ≥15, and a severe sleep‐disordered breathing as an AHI ≥30.^12^ A diagnosis of central sleep apnea was made if the central sleep index was ≥5 and >50% of respiratory events were central.^11^

#### 2.2.1 Statistical analysis

Characteristics of the study population were expressed as medians (with interquartile ranges) and counts (with percentages).

## 3 RESULTS

We identified 17 adults with a diagnosis of DEE who were admitted for 5 days during the monitoring period. In four cases, polysomnography was not performed due to severe behavioral and cognitive challenges. Thirteen adults diagnosed with DEE underwent polysomnography. Six patients had Lennox–Gastaut syndrome, two had Lennox–Gastaut syndrome‐like phenotype, one had Landau–Kleffner syndrome, one had epilepsy with myoclonic‐atonic seizures, and two had unclassified DEEs. Demographic and clinical characteristics are summarized in Table 1. In all patients, routine procedures typically carried our during video‐EEG monitoring including ASM tapering and sleep restriction were not performed during the admission as the purpose of their elective admission was to clarify seizure type and frequency on their current treatment regimen.

**TABLE 1:** Demographic and clinical characteristics of the study participants.

Patient number	Epilepsy syndrome	Age on admission (y)	Sex	Body mass index^a^	Neck circumference (cm)	Antiseizure medications on admission, *n* (%)	Age of onset (y)	Disease duration (y)	Seizure frequency in the month prior to admission	Epilepsy surgery or neuromodulation	MRI findings	Findings of genetic investigations	Comorbidities	Other medications on admission
1	Landau–Kleffner syndrome	21	Female	25.6	32.5	None	5	16	None	None	Small area of gliosis/encephalomalacia in the anterior right thalamus	Karyotype 46,XX, negative of FISH 22q11 microdeletion	Intellectual disability, language regression, anxiety/depression, migraine, coarctation, and VSD repair in infancy	Desvenlafaxine
2	Epileptic encephalopathy^b^	50	Female	45.0	40.0	Carbamazepine, clobazam, lacosamide, and phenytoin	1	49	3–4/d	VNS turned on (2.75 mA, off period of 5 min and stimulation for 30 s)	Left hemiatrophy with increased signal in the left temporal and occipital lobes, right frontal white matter hyperintensity	None	Intellectual disability, hypercholesterolemia	None
3	Epileptic encephalopathy^b^	37	Female	13.3	27.0	Carbamazepine, lamotrigine, and levetiracetam	3 mo	37	1–3/d	None	Dandy‐Walker malformation	None	Severe intellectual disability, Dandy‐Walker syndrome	None
4	Epileptic encephalopathy^b^	27	Male	Not recorded	Not recorded	Carbamazepine	13	14	More than 10/d (not status)	None	Not performed	None	Significant intellectual disability, depression, anxiety, and autism spectrum disorder	Cyproheptadine, fluvoxamine, and olanzapine
5	Lennox–Gastaut syndrome	35	Female	34.5	38.0	Clonazepam, lamotrigine	2.5	32.5	1–6/wk	None	Not performed	None	Severe intellectual disability, Albright's osteodystrophy, VSD closure, and behavioral non‐epileptic events	None
6	Lennox–Gastaut syndrome	28	Female	34.3	34.5	Clobazam, lamotrigine, and topiramate	14	14	>10/d (not status)	None	Not performed	None	Intellectual disability, mosaic chromosome trisomy 15, and chronic idiopathic urticaria	None
7	Lennox–Gastaut syndrome	50	Male	Not recorded	Not recorded	Carbamazepine, lamotrigine, and valproate	2	48	>10/d (not status)	None	Not performed	None	Intellectual disability	None
8	Lennox–Gastaut syndrome	31	Female	22.8	34.0	Levetiracetam, clobazam	24	7	1–3/month	None	Not performed	None	Intellectual disability, Turner's syndrome, type 1 diabetes, and hypertension	None
9	Lennox–Gastaut syndrome	20	Female	22.2	32.0	Clobazam, lacosamide, lamotrigine	9	11	4–10/d	Epilepsy surgery (right frontal craniotomy and corticectomy), age 18	Right frontotemporal neuroepithelial tumor resected at the age of 4	None	Mild intellectual impairment with possible ADHD, left anterior frontotemporal traumatic subdural hemorrhage with evacuation of clot at age 16, hypopituitarism, psychotic disorder	None
10	Lennox–Gastaut syndrome	33	Female	26.9	34.0	Carbamazepine, diazepam	0.4	32.6	1–6/wk	None	Normal	None	Intellectual delay	None
11	Epileptic encephalopathy (Lennox–Gastaut syndrome‐like phenotype)	21	Female	24.0	35.0	Clobazam, lacosamide, perampanel, and topiramate	0.5	20.5	>10/d (not status)	None	Not performed	None	Severe intellectual disability, global developmental delay, cerebral palsy, and asymptomatic hypotension on the background of pyruvate dehydrogenase deficiency	None
12	Late‐onset epileptic encephalopathy (Lennox–Gastaut syndrome‐like phenotype)	31	Male	27.8	38.0	Levetiracetam, zonisamide	11	20	1–3/d	None	Global cerebral atrophy	None	Mild cognitive impairment, some features of cerebellar ataxia, hypercholesterolemia, and hyperammonemia	Benztropine, risperidone
13	Epilepsy with myoclonic‐atonic seizures	23	Male	Not recorded	Not recorded	Lamotrigine, valproate	3.5	19.5	>10/d (not status)	None	Normal	None	Intellectual impairment, social isolation, and major depressive disorder	Medium‐chain triglycerides, Sertraline

Abbreviations: ADHD, attention‐deficit hyperactivity disorder; VNS, vagus nerve stimulation; VSD, ventricular septal defect.

^a^ Body mass index is calculated from the weight in kilograms divided by the square of the height in meters.

^b^ Epileptic encephalopathy not further classified.

### 3.1 Polysomnography findings

Total sleep duration and sleep efficacy ranged from 2.5 to 9.8 h and 50.1%–94.8%, respectively. Moderate‐to‐severe obstructive sleep apnea (OSA; defined as an apnea‐hypopnea index [AHI] of ≥15) was observed in 53.8% (7/13), which was severe (AHI ≥ 30) in 30.8% (4/13). An example of severe OSA in a patient with Lennox–Gastaut syndrome is shown in Figure S1. Polysomnography characteristics are summarized in Table 2.

**TABLE 2:** EEG and polysomnography features of the patients with DEEs.

Case	EEG interictal abnormalities during PSG	Seizures during PSG	AHI	AHI NREM	AHI REM	CSA index	PLM index	N1 min (%)	N2 min (%)	N3 min (%)	REM min (%)	SPT, min	TST, min	Sleep efficacy (%)^a^	WASO (%)	Total arousal index
1	None	None	2.8	3.2	0.8	0	2.5	10	47.6	29	13.4	617.0	586.0	95.0	5.0	12.9
2	Very frequent generalized poly‐spike, spike–wave, and occasional bursts of generalized paroxysmal fast activity	Very frequent electrographic seizures (15.3 seizures/h)	72.6	72.4	78.5	0	2.4	52.6	40.9	2.9	3.6	599.0	359.5	60.0	40.0	55.7
3	Frequent runs of rhythmic delta slowing with bursts of low amplitude poly‐spikes	None	10.6	10.1	24.7	1.9	10.8	4.3	17.4	74.4	3.9	487.5	432.0	88.6	11.4	21.5
4	Frequent, spike–wave, poly‐spike–wave, and poly‐spike discharges	None	21.6	21.6	0.0	0.0	0.0	100.0	0.0	0.0	0.0	177.0	147.5	83.3	16.7	29.3
5	Frequent generalized poly‐spike–wave and spike–wave activity. There are frequent runs of generalized paroxysmal fast activity	None	114.1	117.8	96.2	1.8	0	28.2	40.4	22.6	8.9	519.5	451.0	86.8	13.2	29.0
6	Frequent right temporal spikes and poly‐spikes, which also occurred synchronously over both hemispheres, and bursts of intermittent, high amplitude, generalized paroxysmal fast activity commonly associated a decrease in amplitude to respiration	Frequent tonic seizures (10.4 seizures/h) that commonly occurs with a central apnea	3.8	3.8	0	1.9	5.7	100	0	0	0	587.0	518.0	88.2	11.8	48.9
7	Frequent interictal independent right and left frontal/anterior temporal spikes. Occasional bi‐frontotemporal spikes, with a right frontal predominance	Frequent electrographic and tonic seizures (2.4 seizures/h) that infrequently occur with an obstructive apnea or hypopnea	73.4	69.7	91.3	0.2	20.4	24.8	14.9	45.3	15.0	540.0	353.0	65.4	34.6	65.3
8	Frequent paroxysmal generalized spike–wave and less frequently polyspike–wave activity. Frequent fronto‐temporal spike–wave discharges, predominantly right sided	None	1.4	1.2	2.2	0	88.3	9.3	52.1	20.0	18.6	656.0	592.5	90.3	9.7	13.3
9	Frequent bursts or runs of spike‐ and polyspike‐waves over both hemispheres, with an anterior head predominance. Frequent multifocal single spikes were also seen	Frequent tonic seizures (0.2 seizures/h) with an increase in amplitude to respiration	26.6	27.3	0	3.1	0.0	10.5	36.6	50.3	2.6	629.0	564.5	89.7	10.3	36.0
10	Very frequent generalized spike and wave and polyspike discharges with a posterior quadrant predominance, and runs of generalized paroxysmal fast activity	None	7.9	3.0	56.3	0.0	0.0	15.6	29.5	48.3	6.6	364.5	259.0	71.1	28.9	48.4
11	Frequent bursts or runs of spike‐and poly‐spike‐waves over both hemispheres with an occasional left‐sided predominance	Frequent tonic seizures (4.0 seizures/h) that commonly occurs with a central apnea, and occasionally an obstructive apnea or hypopnea	11.1	11.1	0	6.3	3.6	23.6	70.4	5.7	0.3	586.5	326.0	55.6	44.4	20.2
12	Frequent spike and slow wave and polyspike and slow wave discharges with an occasional left‐sided predominance, runs of rhythmic delta activity with embedded spikes, and runs of generalized paroxysmal fast activity	None	34.1	34	40	0	0	15.1	54.8	29.6	0.5	467.0	398.0	85.2	14.8	4.5
13	Frequent generalized spike and slow wave and generalized paroxysmal fast activity	Frequent tonic seizures (0.4/h) that commonly occurs with a central apnea	19.2	18	30.5	0.4	0	12.3	64	14.5	9.3	593.0	297.0	50.1	49.9	5.1

Abbreviations: AHI, apnea–hypopnea index; CSA, central sleep apnea; NREM, non‐rapid eye movement; PLM, periodic limb movements; REM, rapid‐eye movement; SPT, sleep period time; TST, total sleep time; WASO, wake after sleep onset.

^a^ Sleep efficacy reported as a percentage of TST/SPT. TST is the sum of all sleep stages (min). SPT is defined as the first epoch of sleep to the last epoch of sleep (min).

### 3.2 EEG and polysomnography findings

**FIGURE 1:**
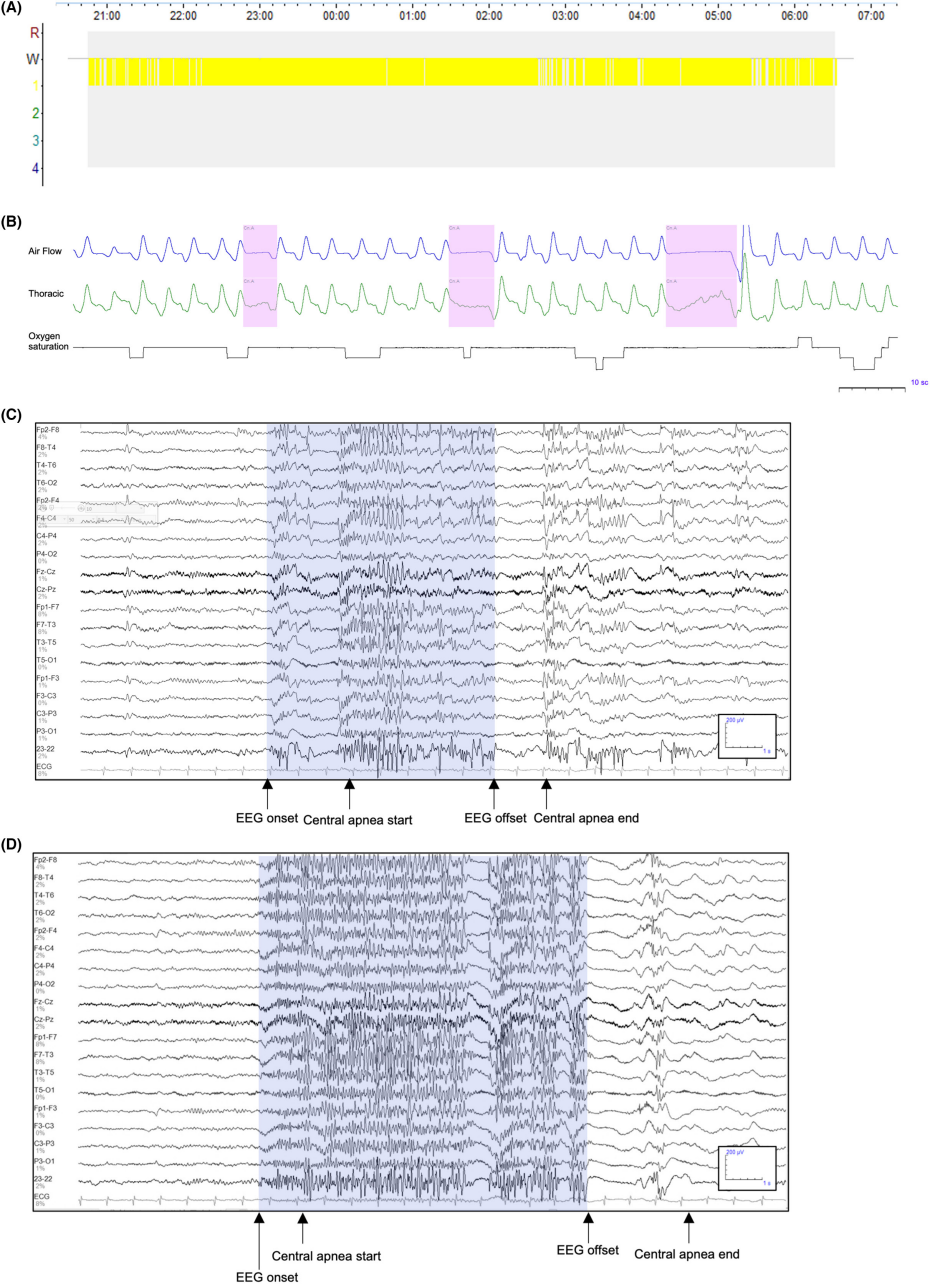
A 28‐y‐old female with Lennox–Gastaut syndrome with highly fragmented sleep architecture (Case 6). There is no evidence of an underlying primary sleep disorder. However, she has frequent epileptiform discharges throughout the recording. In addition, frequent tonic seizures commonly associated with a central apnea and cortical arousal are seen. A, Hypnogram showing a total sleep time duration of 518.0 min, where the patient is only able to achieve stage N1 sleep throughout the entire recording duration. B, Central apneas of 4.9, 6.7, and 10.3 s in duration occur with brief tonic seizures of 5, 7, and 8 s, respectively. There is no change to oxygen saturation for the first apnea, but the second and third are associated with a 3% (nadir 95%) and 4% (nadir 94%) drop in oxygen saturation from baseline. C, EEG recording from a tonic seizure of 6 s associated with a central apnea of 4.9 s, which starts approximately 2 s following the EEG onset. D, EEG recording from a tonic seizure of 8 s associated with a central apnea of 10.3 s, which starts approximately 1 s following the EEG onset.

During the polysomnography recording, 92.3% (12/13) had interictal epileptiform discharges on EEG, and 46.2% (6/13) had seizures which were electrographic in 7.7% (1/13) and electroclinical in 38.5% (5/13). Epileptiform discharges and seizures were commonly associated with microarousals. Three patients had tonic seizures that were commonly associated with central apnea, with an additional patient having infrequent obstructive apnea or hypopnea; the scalp EEG onset preceded the onset of ictal central apnea in all cases. One patient with a Lennox–Gastaut syndrome‐like phenotype and tonic seizures with central apnea met criteria for mild central sleep apnea (Figure S2). Another patient had frequent ictal central apnea during tonic seizures occurring in sleep, which was often the only clinical manifestation (Figure 1). However, this patient did not meet the current guidelines for a diagnosis of central sleep apnea as central apneas were often <10 s.^11^

## 4 DISCUSSION

Our case series demonstrates the effectiveness of polysomnography performed during inpatient video‐EEG monitoring in adults with DEE, which identified a heterogenous pattern of sleep disorders, including a high prevalence of sleep‐disordered breathing. In one patient, we also identified numerous tonic seizures that would have been missed as often their only discernible clinical manifestation was a central apnea.

Over half of our cohort had moderate‐to‐severe OSA. This prevalence was almost twofold greater than that observed in our previous study of epilepsy patients in the same setting (26.3%).^9^ While there have been no previous reports of polysomnography findings in adults with DEEs, small pediatric cohort studies utilizing either polysomnography or overnight oximetry did not observe a high frequency of sleep‐disordered breathing.^1^, ^6^ This suggests that adults with DEEs may be at increased risk of sleep apnea, possibly owing to complications from long‐term use of ASMs. In particular, ASM‐related weight gain may have contributed to the risk of OSA. Three out of four patients with severe OSA had a BMI ≥30; BMI was not recorded in one patient. Benzodiazepines, which were commonly used in our cohort, may also increase the risk of respiratory depression and oxygen desaturation nadir.^13^ Conversely, other ASMs such as carbamazepine, phenobarbitone, valproate, clobazam, clonazepam, phenytoin, and levetiracetam decrease rapid eye movement (REM) sleep^14^; this may underscore the severity of respiratory disturbances in this patient group, which is often exacerbated during REM sleep. Other antiseizure treatments such as vagus nerve stimulation can exacerbate respiratory depression.^15^ There was one VNS‐treated patient in our cohort, who had severe OSA.

Anatomical risk factors including acquired or congenital craniofacial abnormalities such as coarse facies, macroglossia, and bimaxillary protrusion have been reported in DEEs,^16^ and may increase the likelihood of upper airway collapse and obstructive apnea.

Two patients in our study were only able to achieve N1 sleep during the recording period, and most patients failed to achieve adequate REM sleep. This finding is similar to that of a previous report in children with epileptic encephalopathies, where N1 sleep was increased and REM sleep decreased, compared to healthy controls.^6^ The high frequency of sleep apnea and frequent interictal abnormalities and seizures may contribute to increased sleep fragmentation, which may increase seizure likelihood.^17^

Respiratory depression may be more common in tonic seizures due to continuous diaphragm contraction, which may prevent exhalation and cause apnea.^18^ In one patient with frequent tonic seizures, central apnea was often the only clinical manifestation. Many of these events were <10 s, and as such, did not meet criteria for central sleep apnea. However, these brief apneic events are associated with cortical arousal and/or oxygen desaturation, which may be one mechanism for poor sleep quality in this population. Frequent tonic seizures during sleep may represent a risk factor for central sleep apnea in patients with epilepsy, and warrant further investigations.

Our study has limitations. Sleep staging is technically challenging in this patient group due to frequent epileptiform discharges. However, an accredited sleep and neurophysiology scientist and board‐certified epileptologist and sleep/respiratory physician reviewed all investigations. Microarousals occurred with many interictal epileptiform discharges but is difficult to determine whether these changes are causally related or coincidental; the number of clinical seizures may therefore be underreported in our study. We only included patients who were admitted to our video‐EEG monitoring unit for diagnostic evaluation, and as such, these findings may not be generalizable to all patients with DEEs. Further, we only included patients who cooperated with polysomnography and our findings may not be generalizable to those with more severe behavioral and cognitive impairment who likely have more severe polysomnographic abnormalities. As polysomnography was only performed on one night, we were unable to study the effect of nocturnal seizures from the night prior and the impact that this may have on sleep quality. Some patients were on concomitant antipsychotics and/or antidepressants which may affect sleep architecture and respiratory depression. Further, the findings are derived from polysomnography performed during inpatient video‐EEG monitoring, which may not reflect habitual sleep patterns. However, none of our patients underwent ASM tapering or sleep restriction procedures or were given seizure rescue medications during the admission.

## 5 CONCLUSION

This study identified a high prevalence of sleep abnormalities in adults with DEEs. Disrupted sleep may increase the risk of epileptiform discharges and seizures, and conversely, seizures and epileptiform discharges may contribute toward sleep disruption in this population. Further studies should examine whether suppression of seizures and epileptiform discharges improves sleep in DEE patients. To overcome the practical challenge in performing polysomnography in this patient population and to capture habitual sleep patterns, the validity of using novel technologies such as wearable devices for sleep assessment should be investigated. Respiratory monitoring identified a number of clinical seizures that would have otherwise been missed and may improve the diagnostic yield of video‐EEG monitoring.

## AUTHOR CONTRIBUTIONS

Dr. Shobi Sivathamboo: Conceptualized and designed the study, drafted and revised the article for intellectual content, reviewed clinical data, analyzed data, contributed to the interpretation of data, and prepared the tables and figures. Dr. Kenneth A. Myers: Critical revision of the article for intellectual content. Dr. Andreas Pattichis: Analyzed data and critical revision of the article for intellectual content. Ms. Elise J. White: Analyzed data and revised the article for intellectual content. Ms Ka Nyuk Ku: Analyzed data and revised the article for intellectual content. Dr. Terence J. O'Brien: Critical revision of the article for intellectual content. Dr. Piero Perucca: Critical revision of the article for intellectual content. Patrick Kwan: Provided scientific direction and critical revision of the article for intellectual content.

## FUNDING INFORMATION

This work was supported by a Medical Research Future Fund Practitioner Fellowship (MRF1136427), as well as a Program Grant (APP1091593) and Investigator Grant (APP1176426) from the National Health and Medical Research Council of Australia. The funders had no role in the design and conduct of the study; collection, management, analysis, and interpretation of the data; preparation, review, or approval of the article; and decision to submit the article for publication.

## CONFLICT OF INTEREST

We confirm that we have read the Journal's position on issues involved in ethical publication and affirm that this report is consistent with those guidelines.

## STANDARD PROTOCOL APPROVALS, REGISTRATIONS, AND PATIENT CONSENTS

Polysomnography was performed as part of routine clinical care. As such, the Human Research Ethics Committee of Melbourne Health and Alfred Health approved the study with a waiver of informed consent.

## Supporting information


Figure S1.
Figure S2.

## Data Availability

Anonymized participant data will be shared by request from any qualified researcher.
