# Supplementary material for: Sleep and respiratory abnormalities in adults with developmental and epileptic encephalopathies using polysomnography and video‐EEG monitoring
Source: Epilepsia Open. 2023 Jun 12;8(3):1157–68. doi: 10.1002/epi4.12772 (PMC10472408; doi:10.1002/epi4.12772)
Supplement: Supplementary file 1 — Figure S1. Figure S2. [file EPI4-8-1157-s001.docx]

**SUPPLEMENTARY FIGURES**

**Figure S1.** Polysomnogram recording from a 50-year-old female with Lennox-Gastaut syndrome and severe obstructive sleep apnea (Patient 2). This patient has an apnea-hypopnea index of 72.6 per hour. (a) Frequent obstructive apneas are seen that are 10.3s, 22.2s, 59.0s, 17.7s, 49.6s, and 44.5s in duration (purple boxes). There is suppression of airflow and respiratory effort in both thoracic and abdominal channels. This patient has a vagal nerve stimulator implanted with an output current of 2.75 mA, which may cause respiratory depression during stimulation. However, the frequency of stimulation is every 5 minutes for 30 seconds, which cannot alone explain the severity of sleep-disordered breathing.

F7-T3

F3-C3

P3-O1

F4-C4

C4-P4

F8-T4

Right Eye

Left Eye

Chin

Snore

ECG

Position

Nasal Pressure

Thoracic

Abdominal

Oxygen Saturation

Pulse Rate

Left Leg

Right Leg


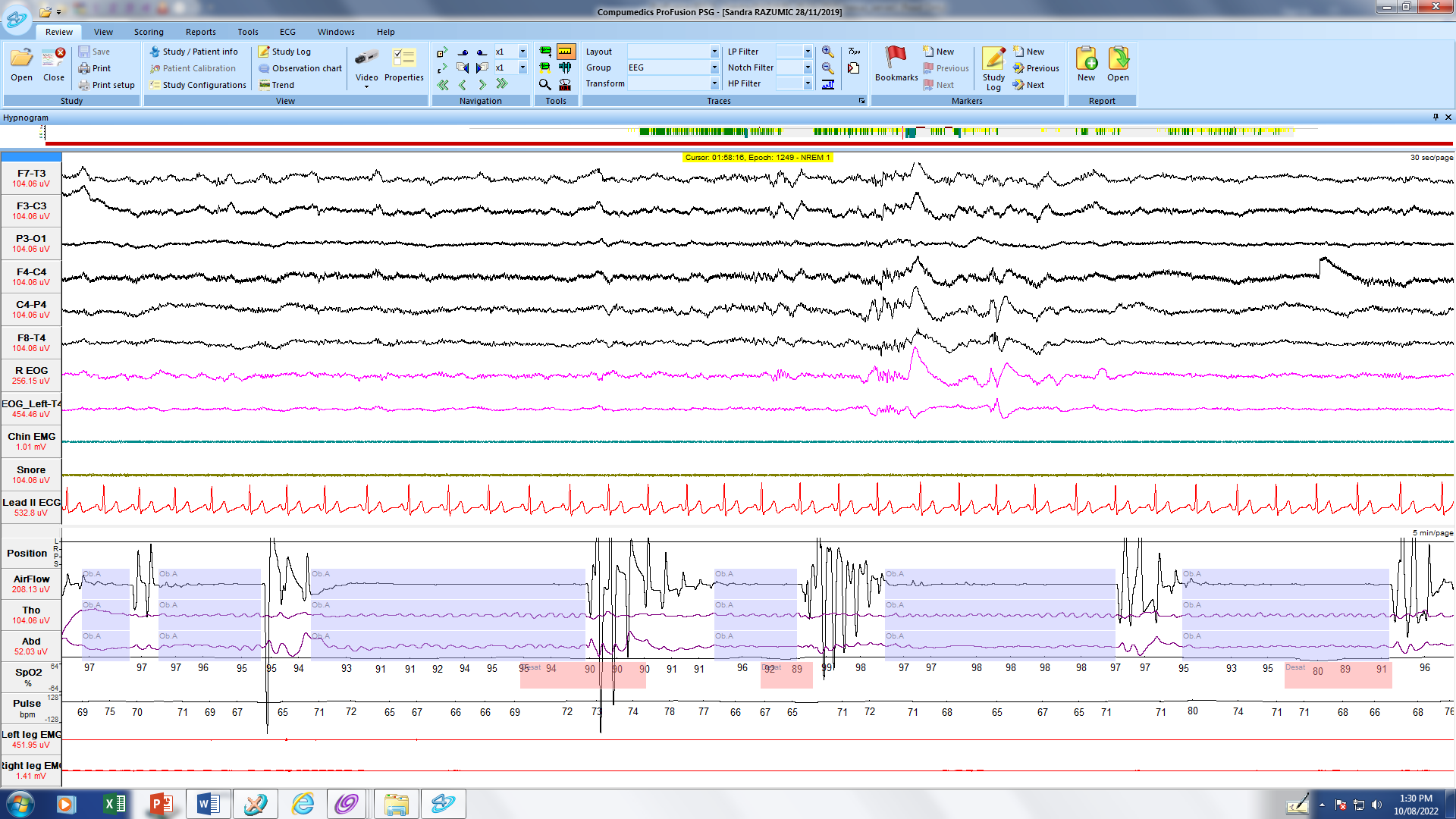

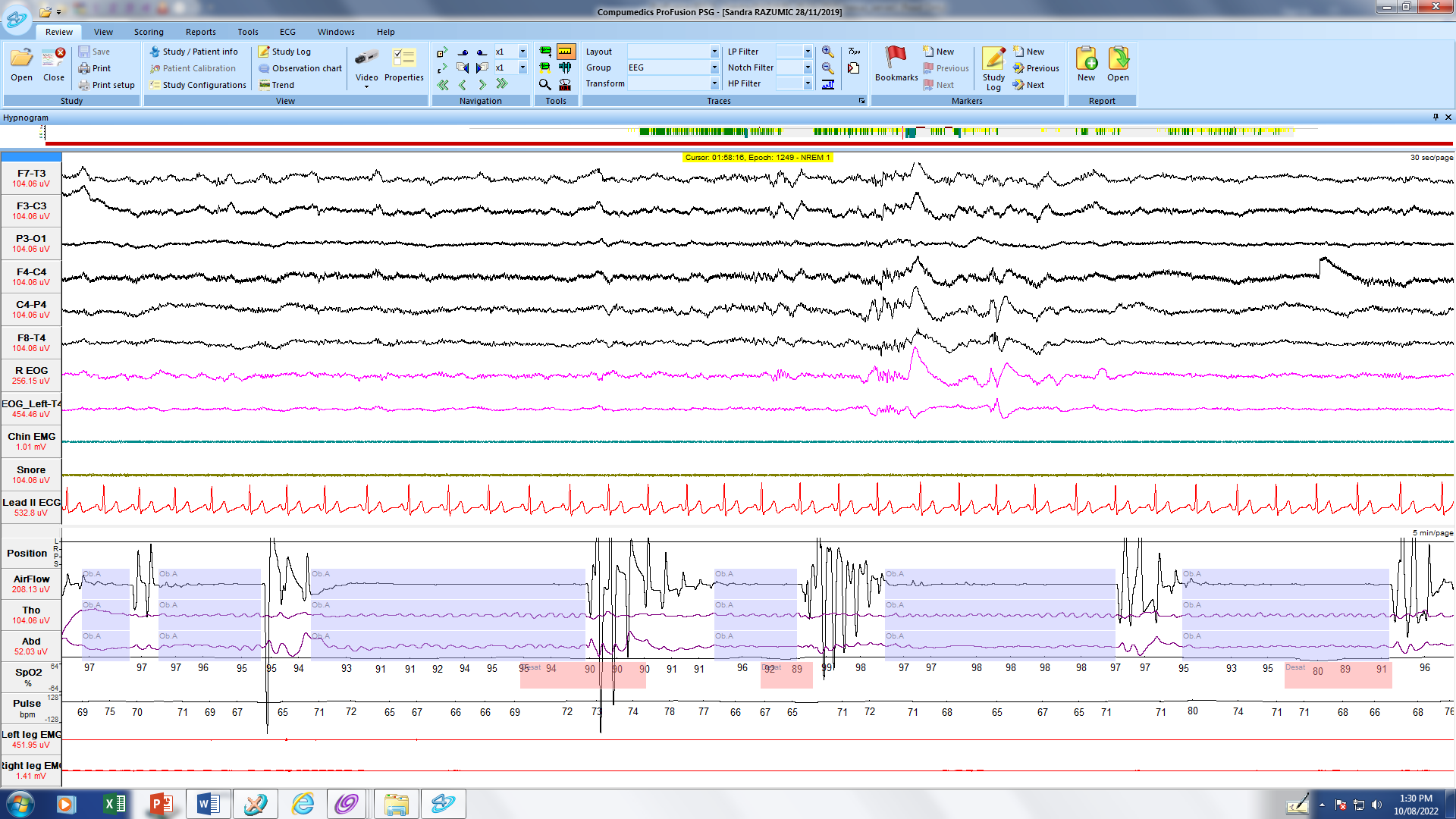


**Figure S2.** A 21-year-old patient with a Lennox-Gastaut syndrome-like phenotype with frequent tonic seizures (4.0/hour) and central apnea (Patient 11). (A) EEG recording showing the unequivocal EEG onset, which is associated with a central apnea two seconds later. (B) The polysomnogram recording showing an initial reduction in the amplitude of the airflow signal, which progresses to a central apnea that lasts 21 seconds and terminates 6 seconds following the EEG offset (pink box). This is accompanied with a 5% drop in oxygen saturation (nadir 93%). The overall apnea-hypopnea index is mild at 11.1 events/hour. However, the patient has a central sleep index of 6.3 events/hour, which is consistent with a diagnosis of central sleep apnea secondary to tonic seizures.

EEG onset

Central apnea start


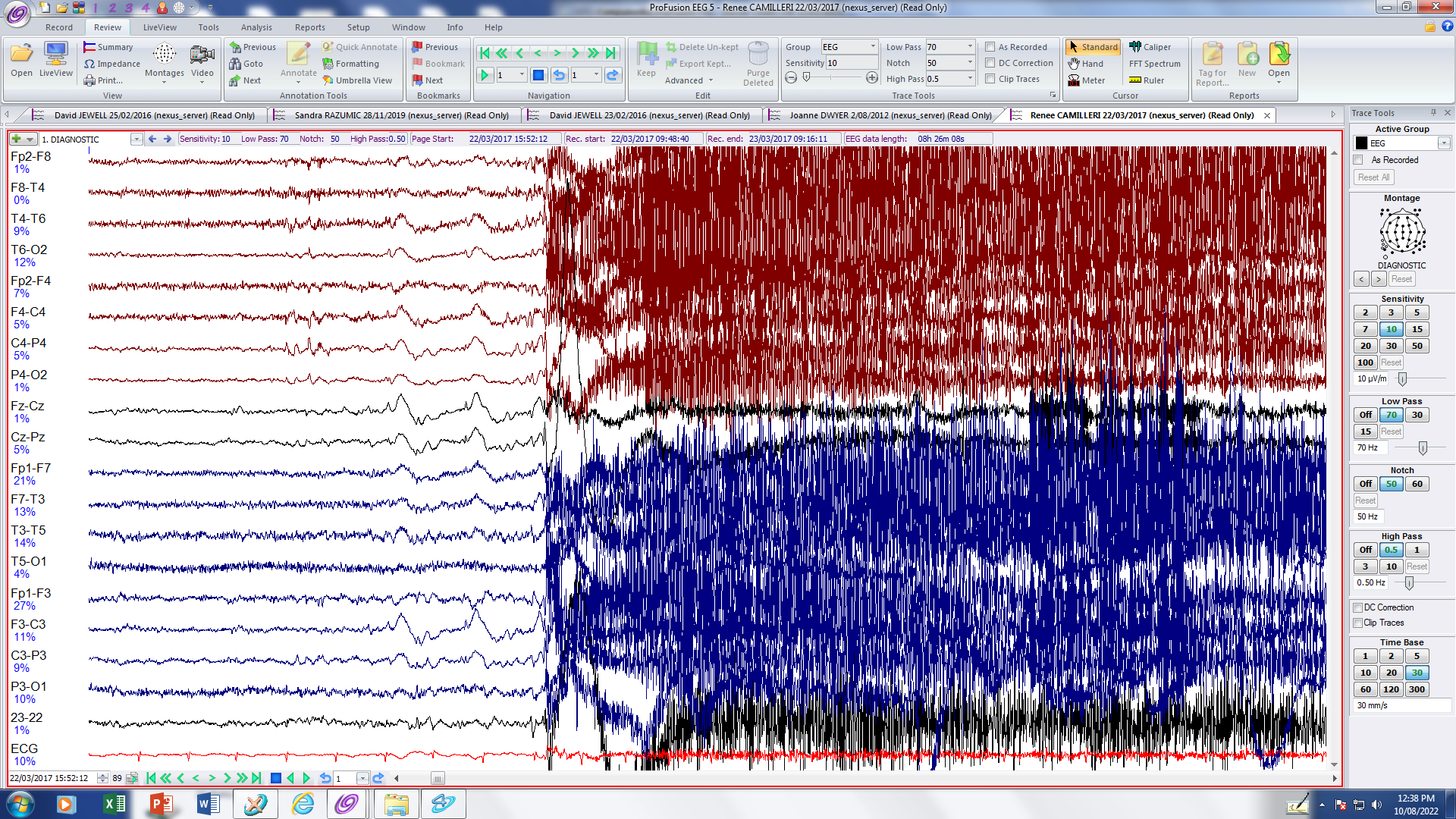

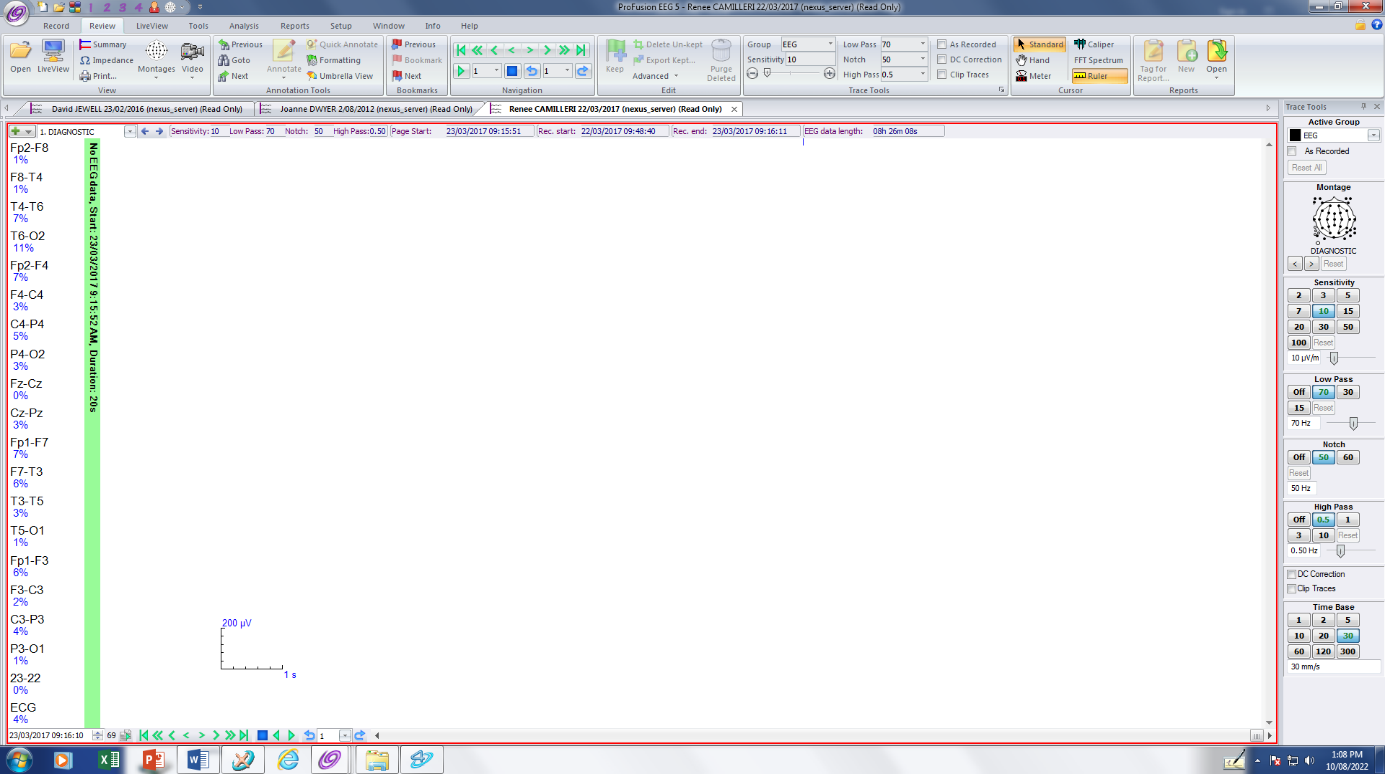

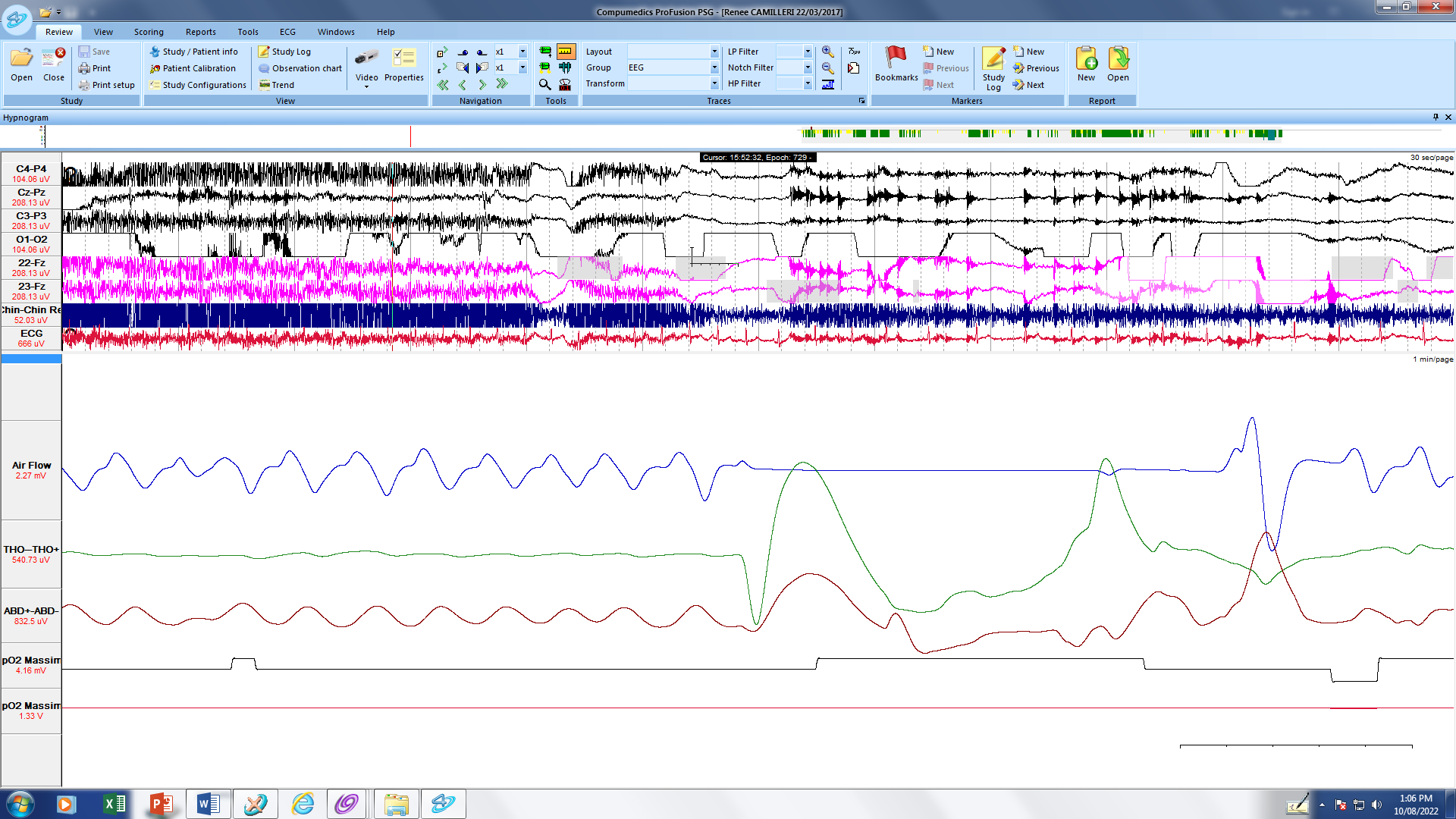


Air Flow

Thoracic

Abdominal

Oxygen saturation


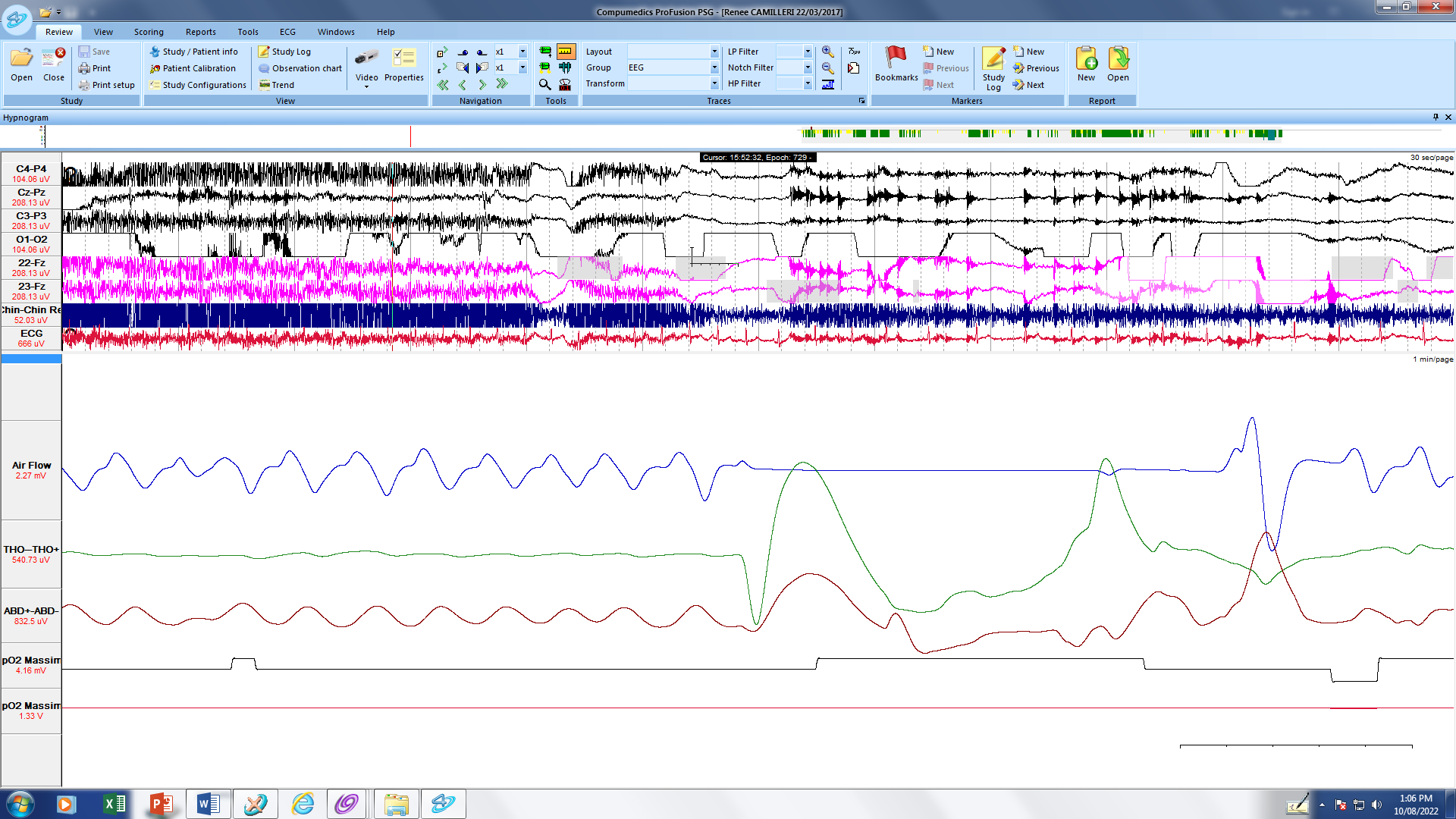


Central apnea start

10 sc

Central apnea end

A

B
